# Neotropical Echinococcosis Complicated by Hepatic Abscess and Hepato-Pleural Fistula: A Case Report From the State of Acre, Brazil

**DOI:** 10.7759/cureus.97235

**Published:** 2025-11-19

**Authors:** Nilton G Siqueira, Viktor D Magalhães, Thor O Dantas, Everton G Beltrame, Marco Antonio C Guedes

**Affiliations:** 1 School of Medicine, Federal University of Acre, Rio Branco, BRA; 2 Surgery Department, Acre Clinical Hospital, Rio Branco, BRA; 3 Urology Department, Base Hospital, Faculty of Medicine of São José do Rio Preto, São José do Rio Preto, BRA; 4 Infectious Disease and Hepatology Department, Acre Clinical Hospital, Rio Branco, BRA; 5 Thoracic Surgery Department, Emergency and Urgency Hospital of Rio Branco, Rio Branco, BRA; 6 Surgery Department, Santa Marcelina Hospital, Porto Velho, BRA

**Keywords:** echinococcus vogeli, hepato-pleural fistula, neotropical echinococcosis, polycystic hydatidosis, western amazon

## Abstract

Polycystic echinococcosis is a parasitic disease caused by cestodes of *Echinococcus vogeli*, which occurs in rural and wild areas of Central and South America. Its definitive hosts are *Speothos venaticus* (bush dog) and *Canis familiaris* (domestic dog), while *Cuniculus paca* (paca) serves as the intermediate host. Humans become accidental hosts by consuming food or water contaminated with eggs of this helminth. In the Amazon, due to geographic, economic, and cultural factors, rural populations often consume wild meat as a main protein source. The viscera of pacas are used to feed domestic dogs, perpetuating the life cycle of *E. vogeli*. We describe a rural patient with hepatitis B virus (HBV)-related cirrhosis and neotropical echinococcosis who developed a hepatic abscess that fistulized into the right hemithorax. Due to the low hepatic reserve and extensive liver involvement, the conventional surgical approach for echinococcosis was contraindicated.

## Introduction

Echinococcosis is a disease caused by cestodes of the genus *Echinococcus*, which includes nine distinct species: *E. granulosus*, *E. canadensis*, *E. ortleppi*, *E. felidis*, *E. equinus*, *E. multilocularis*, *E. shiquicus*, *E. oligarthra*, and *E. vogeli* [1]. The last two are responsible for neotropical echinococcosis, restricted to Central and South America, particularly the Amazon region [2,3]. The biological cycle of *E. vogeli* involves infected pacas (*Cuniculus paca*) and agoutis (*Dasyprocta aguti*) [4]. In the state of Acre, Brazil, rural inhabitants with low socioeconomic status often consume wild animal meat, mainly paca. The viscera are commonly given to domestic dogs, which become definitive hosts of the parasite. Humans, as accidental hosts, acquire the infection by ingesting food or water contaminated with eggs excreted in dog feces [5]. The aim of this article is to demonstrate less invasive and more effective surgical management options for critically ill patients with low hepatic functional reserve.

This article was previously presented as a poster at the XXXIII Brazilian Congress of Surgery in 2019 in Brasília, Brazil.

## Case presentation

**Figure 1:**
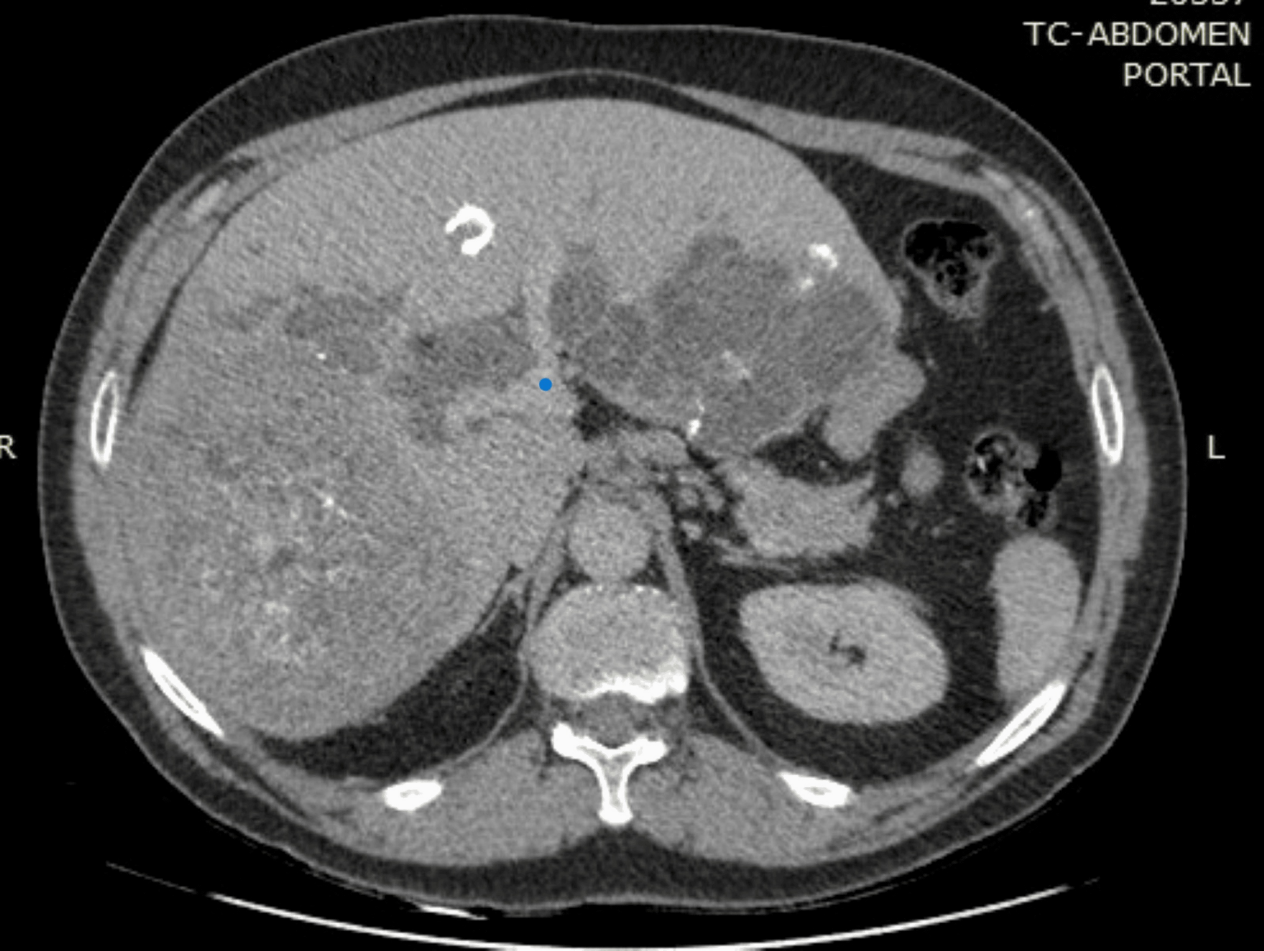
Computed tomography from March 2018 demonstrating multiple cystic lesions with typical coarse calcifications in both lobes, encompassing the portal vein (blue dot).

**Figure 2:**
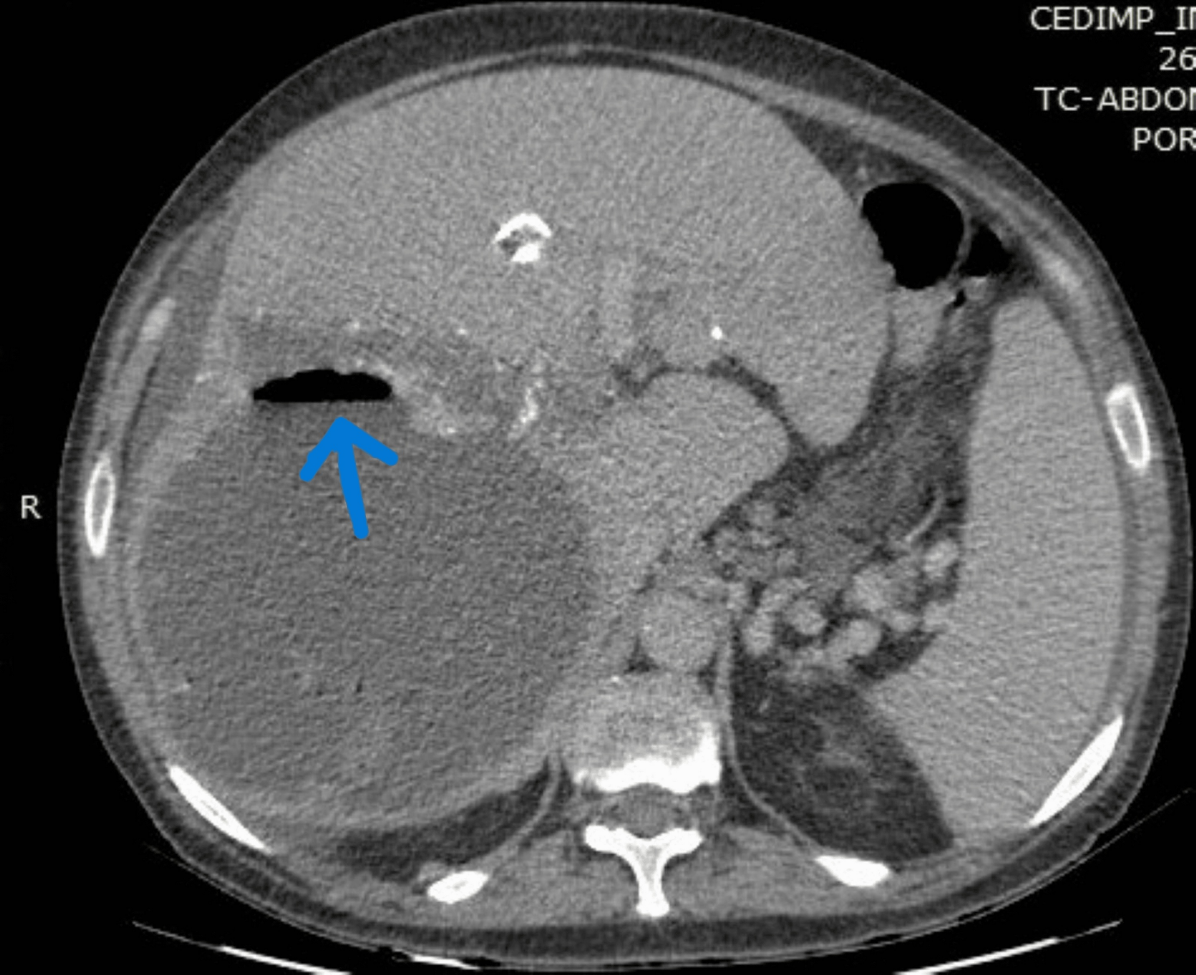
Computed tomography scan during hospitalization showing right lobe air-fluid level (blue arrow).

A 53-year-old male farmer with hepatitis B virus (HBV)-related cirrhosis (Child-Pugh B) and neotropical echinococcosis, under treatment with entecavir and albendazole (Figure 1), presented with right upper quadrant pain radiating to the back, anorexia, and fever. Computed tomography (CT) revealed multiple cystic lesions coalescing into an abscess occupying the right hepatic lobe (Figure 2).

**Figure 3:**
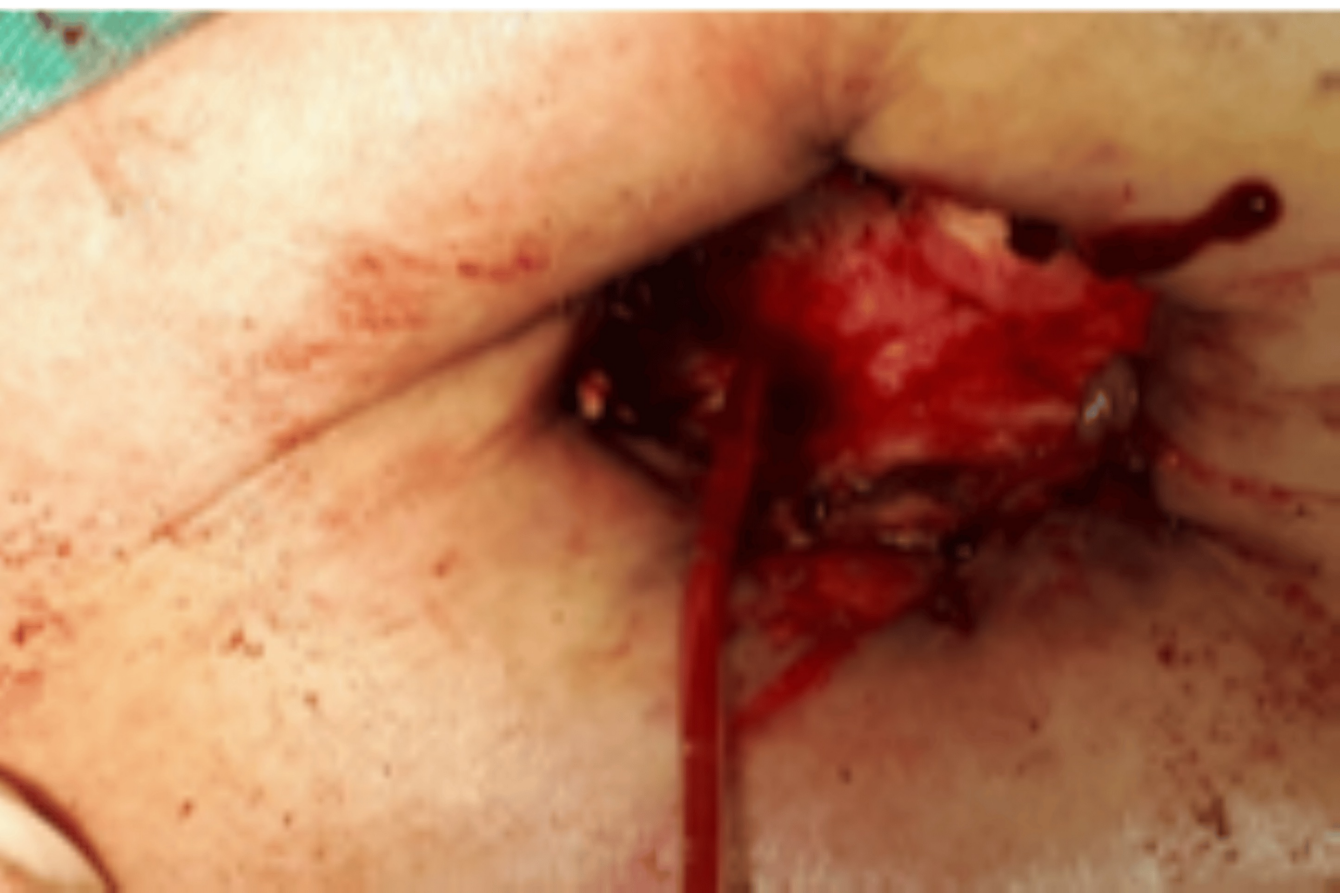
Pleurostomy. Catheterization of a fistulous hole between the hepatic dome and the pleural cavity during the preparation of the pleurostomy.

He received imipenem for 21 days and subsequently underwent percutaneous drainage, yielding 750 mL of purulent material. Postoperatively, he developed chest pain, a dry cough, and dyspnea. CT imaging showed a large right pleural empyema, which was drained via a water-seal system with high output for 15 days. Due to partial lung entrapment, open drainage with saline irrigation and respiratory physiotherapy was performed. Subsequent CT showed persistent pulmonary encasement and a new abscess at the posterior base of the right hemithorax. Pleurostomies were performed bilaterally under local anesthesia and sedation, revealing a diaphragmatic orifice corresponding to a hepato-thoracic fistulous tract feeding the empyema, indicating contiguous spread (Figure 3). ​​​​​​

**Figure 4:**
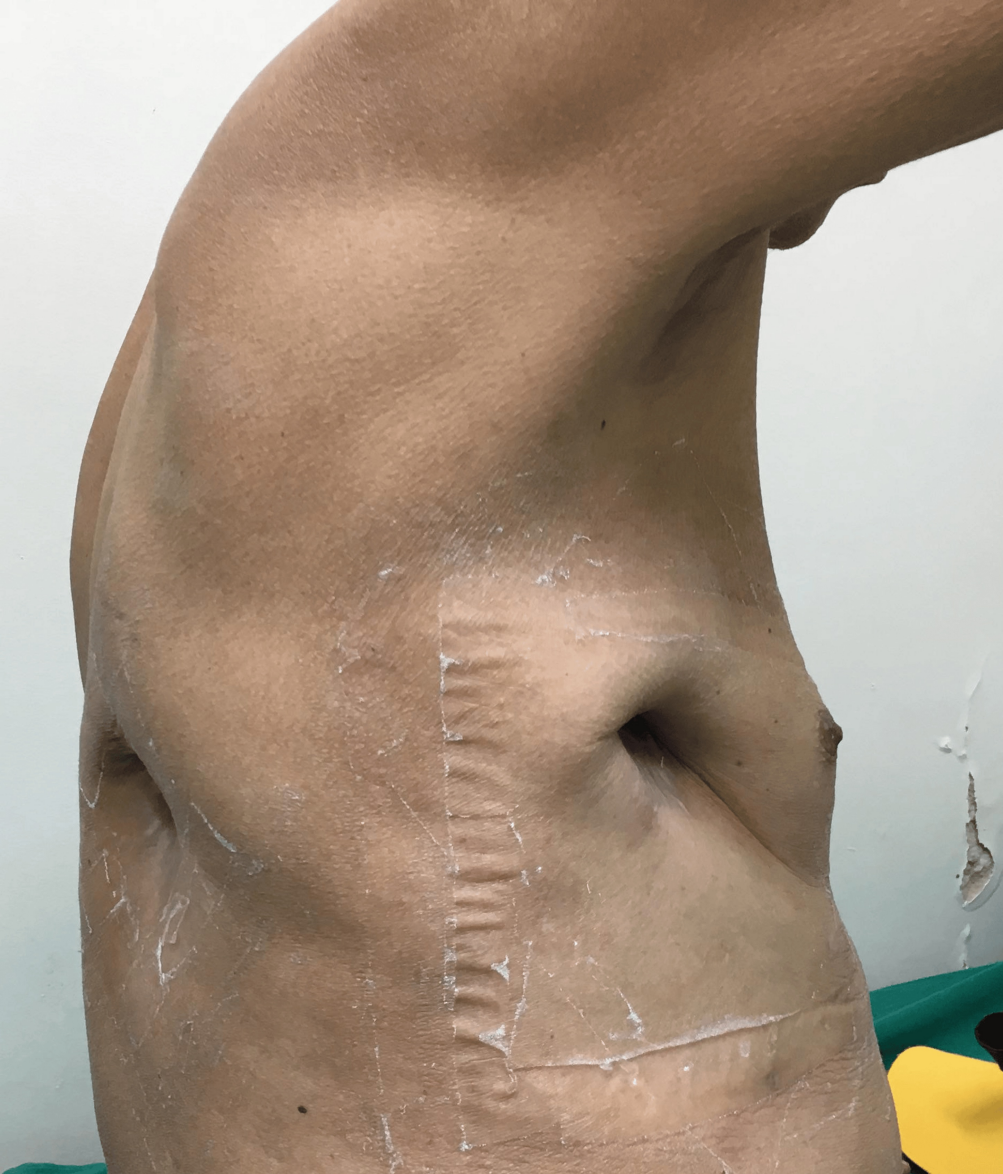
Return to the outpatient clinic. Seventy-six days after pleurostomies, there were minimal residual cavities and no secretion.

The patient developed severe anemia, malnutrition, ascites, and lower limb edema, treated with blood transfusions, albumin, diuretics, paracentesis, and enteral nutritional support. After progressive clinical improvement, he was discharged after 88 days (Figure 4).

**Figure 5:**
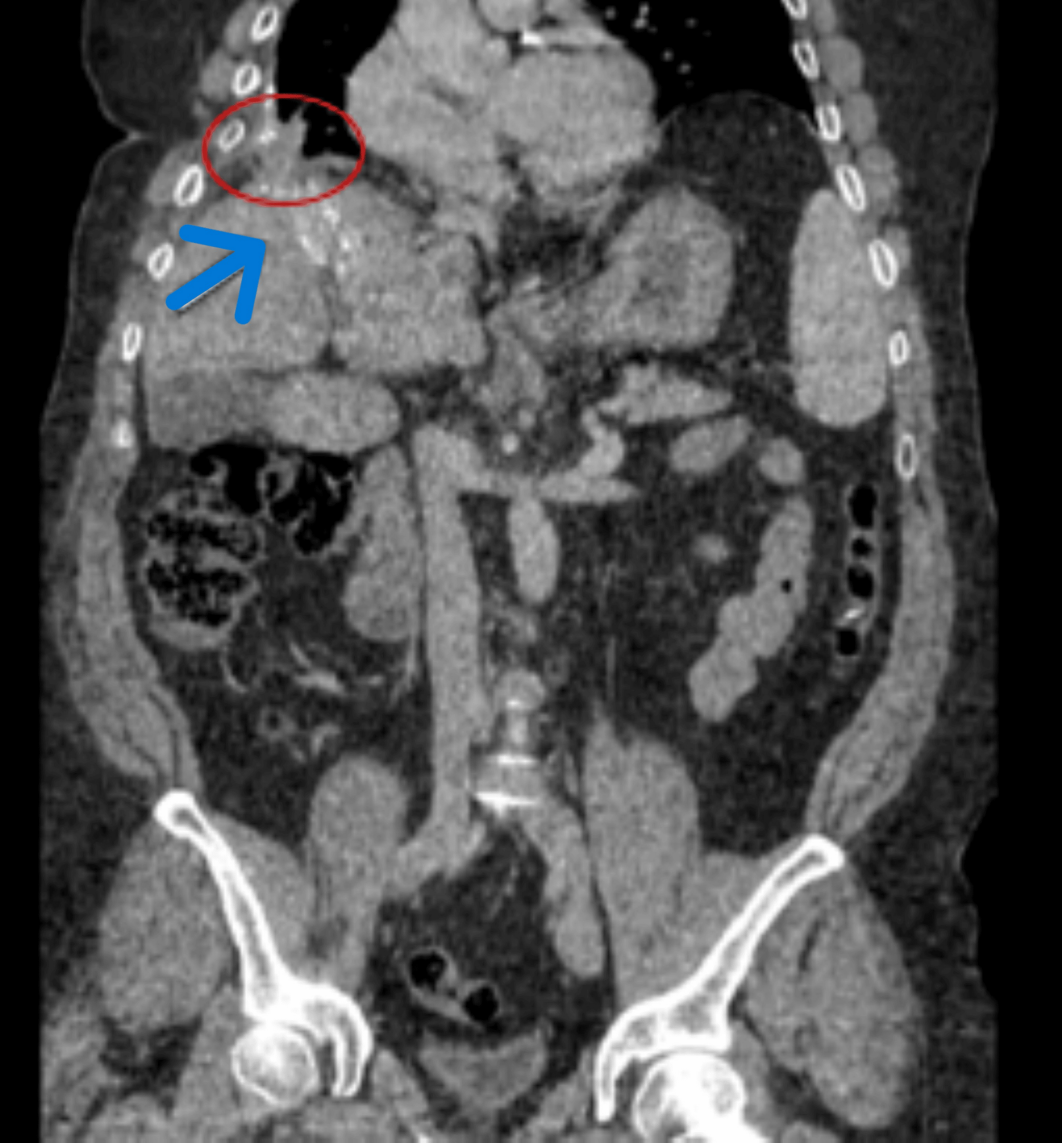
Computed tomography scan of control after six years demonstrating residual hepatic calcifications (blue arrow) and fibrosis resulting from hepato-pleural fistula (within the red circle) and absence of recurrence.

Six years later, he remains in good general condition, with follow-up CT showing residual hepatic calcifications and fibrosis from the hepato-pleural fistula (Figure 5).​​​​​

## Discussion

Clinical manifestations of echinococcosis depend on the affected organ and are often asymptomatic. The liver is involved in approximately 80% of cases, followed by the lungs and abdominal cavity. Symptoms arise when cysts exert extrinsic compression on adjacent structures [4]. Hepatic echinococcosis complications include abscess formation, biliary fistula, and hepato-thoracic communication when lesions are contiguous with the diaphragm, occurring in 2-11% of cases [6,7]. Diagnosis relies on epidemiologic history, physical examination, radiologic imaging, and laboratory tests. Ultrasonography is the preferred diagnostic method in endemic areas due to its low cost and portability, despite being operator-dependent. CT plays an essential role in diagnosis, prognosis, and treatment planning. Treatment depends on disease stage and imaging classification and may include the following: (1) surgery, (2) PAIR (puncture, aspiration, injection of scolicidal agent, re-aspiration), (3) medical therapy with benzimidazoles, or (4) "watch and wait". Surgery is the most common and effective approach and generally leads to a cure. New surgical techniques have facilitated complete resection even in advanced cases. Thoracotomy or thoracophrenolaparotomy may be indicated when hepatic and pulmonary echinococcosis coexist, although this remains controversial. Liver transplantation may also be an option [8]. In this case, surgery was contraindicated due to poor hepatic reserve. The coexistence of HBV-related cirrhosis adds complexity, as these patients have compromised immunity, hypoalbuminemia, and ascites. Cirrhotic patients are prone to severe bacterial infections, including cyst infection and abscess formation, which, after drainage and infection control, may also resolve echinococcal infection [9]. Early diagnosis and management are crucial to prevent severe and potentially fatal complications. The management of patients with low liver reserve should include less invasive techniques in order to maintain homeostasis as close to normal.

## Conclusions

Neotropical echinococcosis remains a neglected zoonosis in the Amazon region. Awareness among healthcare professionals is vital for early recognition and treatment. Patients with concurrent liver disease, especially HBV-related cirrhosis, present additional management challenges. Early diagnosis, appropriate antimicrobial therapy, and multidisciplinary follow-up can prevent life-threatening outcomes. Interrupting the human cycle by avoiding feeding domestic dogs raw and contaminated viscera is fundamental to controlling this disease, which remains underdiagnosed and unknown to much of the scientific community.
